# The role of small in-frame insertions/deletions in inherited eye disorders and how structural modelling can help estimate their pathogenicity

**DOI:** 10.1186/s13023-016-0505-0

**Published:** 2016-09-14

**Authors:** Panagiotis I. Sergouniotis, Stephanie J. Barton, Sarah Waller, Rahat Perveen, Jamie M. Ellingford, Christopher Campbell, Georgina Hall, Rachel L. Gillespie, Sanjeev S. Bhaskar, Simon C. Ramsden, Graeme C. Black, Simon C. Lovell

**Affiliations:** 1 Manchester Royal Eye Hospital, Manchester Academic Health Science Centre, Manchester, UK; 2 Centre for Ophthalmology & Vision Sciences, University of Manchester, Manchester, UK; 3 Manchester Centre for Genomic Medicine, St Mary’s Hospital, Manchester Academic Health Sciences Centre, Manchester, UK; 4 School of Biological Science, University of Manchester, Manchester, UK

**Keywords:** Inherited eye disease, Retinal dystrophy, Childhood cataract, In-frame insertions/deletions, Homology modeling

## Abstract

**Background:**

Although the majority of small in-frame insertions/deletions (indels) has no/little affect on protein function, a small subset of these changes has been causally associated with genetic disorders. Notably, the molecular mechanisms and frequency by which they give rise to disease phenotypes remain largely unknown. The aim of this study is to provide insights into the role of in-frame indels (≤21 nucleotides) in two genetically heterogeneous eye disorders.

**Results:**

One hundred eighty-one probands with childhood cataracts and 486 probands with retinal dystrophy underwent multigene panel testing in a clinical diagnostic laboratory. In-frame indels were collected and evaluated both clinically and *in silico*. Variants that could be modeled in the context of protein structure were identified and analysed using integrative structural modeling. Overall, 55 small in-frame indels were detected in 112 of 667 probands (16.8 %); 17 of these changes were novel to this study and 18 variants were reported clinically. A reliable model of the corresponding protein sequence could be generated for 8 variants. Structural modeling indicated a diverse range of molecular mechanisms of disease including disruption of secondary and tertiary protein structure and alteration of protein-DNA binding sites.

**Conclusions:**

In childhood cataract and retinal dystrophy subjects, one small in-frame indel is clinically reported in every ~37 individuals tested. The clinical utility of computational tools evaluating these changes increases when the full complexity of the involved molecular mechanisms is embraced.

**Electronic supplementary material:**

The online version of this article (doi:10.1186/s13023-016-0505-0) contains supplementary material, which is available to authorized users.

## Background

Small insertions/deletions (indels) are the second most abundant form of human genetic variation after single nucleotide variants (SNVs) [1]. These DNA changes can influence gene products through multiple mechanisms, including altering amino acid sequence and affecting gene expression [2]. A number of computational tools that functionally annotate indels are available including SIFT-indel [3], PROVEAN [4], DDG-in [5], CADD [6], PriVar [7], PinPor [2], HMMvar [8], KD4i [9], and VEST-indel [10]. Although some of these tools are reported to achieve relatively high sensitivity and specificity values [10], predicting the effect of protein-coding (frameshifting, in-frame) and non-protein-coding indels in the clinical setting remains a formidable challenge [11].

Inherited eye disorders such as childhood cataracts (CC) and retinal dystrophies (RD) are a major cause of blindness among children and working-age adults [12, 13]. Over the past decades, exciting progress has been made in elucidating the genetic basis of these disorders. Hundreds of disease-causing genes have been identified leading to the development of diagnostic tests that are now regularly used in clinical practice [14, 15]. The preferred testing method at present is panel-based genetic diagnostic testing [16], although whole genome sequencing is increasingly being used in the clinical domain [17]. For these tests to have the greatest medical impact, it is necessary to be able to pinpoint the disease-causing variant(s) among the considerable background of detected rare changes that might be potentially functional but not actually responsible for the phenotype under investigation [18]. Guidelines for assigning clinical significance to sequence variants have been developed [19] and it is clear that, among protein-coding changes, in-frame indels present a unique challenge.

When the phenotypic relevance of a protein-coding variant is investigated, knowledge of the structure and biochemistry of the associated protein can be very useful. Unfortunately, due to limitations of mainstream structural biology techniques (X-ray crystallography [XRC], nuclear magnetic resonance [NMR], 3D electron microscopy [3DEM]), experimentally determined structures are available for only a small proportion of proteins [20]. Recently, computational methods have been used to generate reliable structural models based on complementary experimental data and theoretic information [21]. Such integrative modeling approaches can be utilised to evaluate protein-coding variants *in silico*, on the basis of 3D structure and molecular dynamics [22].

In this study, a variety of methods including integrative modeling, are used to gain insights into the role of in-frame indels in two genetically heterogeneous Mendelian disorders, CC and RD. Clinical genetic data (multigene panel testing) from 667 individuals are presented and 17 previously unreported in-frame indels are described.

## Methods

### Clinical samples

Unrelated subjects with inherited eye disorders were retrospectively ascertained through the database of the Manchester Regional Genetic Laboratory Service, Manchester, UK. Referrals were received between October 2013 and December 2015 from multiple clinical institutions in the UK and around the world, although a significant proportion of samples came from the North West of England. After obtaining informed consent from the affected individual/family, the referring physician requested a multigene panel test. The reason for referral was included in the clinical data completed by the referring medical specialist. Extensive phenotypic information was available for subjects referred from the Central Manchester University Hospitals, Manchester, UK. Ethics committee approval was obtained from the North West Research Ethics Committee (11/NW/0421 and 15/YH/0365) and all investigations were conducted in accordance to the tenets of the Declaration of Helsinki.

### Genetic and bioinformatic analysis

Testing and analysis were performed at the Manchester Regional Genetic Laboratory Service, a United Kingdom Accreditation Service (UKAS) - Clinical Pathology Accredited (CPA) medical laboratory (CPA number 4015). DNA samples were processed using Agilent SureSelect (Agilent Technologies, Santa, Clara, CA, USA) target enrichment kits designed to capture all exons and 5 base pairs (bp) of the flanking intronic sequence of either

(i) 114 genes associated with CC and/or anterior segment developmental anomalies [14] or
(ii) 176 genes associated with RD.

The genes were selected after interrogating publically available databases (http://cat-map.wustl.edu and http://sph.uth.edu/retnet/) and the literature. A list of all the tested transcripts/genes can be found in Additional file 1: Table S1.

After enrichment, the samples were sequenced on an Illumina HiSeq 2500 system (Illumina Inc, San Diego, CA, USA; 100 bp paired-end reads) according to the manufacturer’s protocols. Sequence reads were subsequently demultiplexed using CASAVA v1.8.2 (Illumina Inc, San Diego, CA, USA) and aligned to the hg19 reference genome using the Burrows Wheeler Aligner (BWA-short v0.6.2) [23]. Duplicate reads were removed using Samtools before base quality score recalibration and indel realignment using the Genome Analysis Tool Kit (GATK-lite v2.0.39) [24]. The UnifiedGenotyper within GATK was used for SNV and indel discovery [25]; indels supported by <0.1 of the reads were discarded and the quality metrics for keeping SNVs included read depth ≥50x and mean quality value (MQV) ≥45.

Previous studies have shown that the number of indels called has a significant positive correlation with the coverage depth [26–28]. Therefore, only samples in which ≥99.5 % of the target region was covered to a minimum depth of 50x were included.

Variant annotation and clinical variant interpretation was performed as previously described [14, 15]. Briefly, the Ensembl Variant Effect Predictor (VEP) was used to assign functional consequences to SNVs and indels. Variants with allele frequency >1 % in in large publically available datasets (National Heart, Lung, and Blood Institute Exome Sequencing Project Exome Variant Server ESP6500 and dbSNP v135) were deemed benign and were not analysed further. The remaining changes were assigned a pathogenicity classification score according to previously described methods [14, 15, 19]. Variants that were suspected to be pathogenic or relevant were included in a clinical report (“clinically reported’), while all other rare changes were included in a technical report. Certain flagged cases were reviewed in a monthly multidisciplinary team (MDT) meeting who discussed in detail the family history, phenotypic presentation and relevant pathogenicity of the identified variants [15]; the decision to include a change in the clinical or technical report was not altered by the MDT. All clinically reported SNVs and indels, and all indels that were novel to this study (i.e. not previously described in Ensembl VEP v83) were confirmed by Sanger sequencing; no false positives were detected. On a few occasions, samples from family members were also analyzed with Sanger sequencing.

### Small insertion/deletion analysis

There is no consensus in the literature about the size range of a ‘small indel’ and, here, we define it as a gain or loss of ≤21 nucleotides at a single locus [2]. There are two reasons for this choice. First, when the Illumina short-read sequencing platform is used, available bioinformatics tools can only detect relatively small indels [28]. Importantly, the sensitivity of such tools is greatly reduced for variants >21 bp [29]. Second, there is evidence to suggest that indels of length ≤21 bp make up the vast majority of all indel events, especially exonic ones [1, 30, 31].

Small in-frame indels were collected and manually checked for redundancy with respect to variants already in Ensembl Release 83 (accessed 03 Mar 2016). Furthermore, changes within 2 bp from intron-exon boundaries were sought after. Indels were then classified based on their primary sequence context into homopolymer runs (HR; if the variant was within a run of six or more identical bases) and tandem repeats (TR; if the variant was within a segment of at least two repeated sequences) [30]. *In silico* analysis using the SIFT-indel [3], PROVEAN [4] and DDG-in [5] computational tools was subsequently performed (all accessed 03 Mar 2016). These three tools were selected as they were freely available at the time of the study design, they have been shown to have high accuracy (>0.80), and they are among the most widely used methods in the field [10].

Integrative protein structure modeling was attempted for all proteins found to harbour small in-frame indels. Reference amino acid sequences (obtained from UniProt) were used to ‘search by sequence’ in the RCSB Protein Data Bank (PDB; accessed 03 Mar 2016) [32]; the BLAST method and an E-value cutoff of 10^−3^ were used.

Manual inspection of the generated alignments was subsequently performed. A prerequisite for reliable integrative modeling is amino acid sequence similarity between the experimentally determined structural model and the input protein. For the purposes of the present study, the area around the mutated locus is of particular importance. Therefore, only cases with >5/11 sequence identity in the part of the alignment that included the variant locus and 5 flanking residues on either side were selected. We note that there is no consensus on what constitutes sufficient sequence similarity for reliable integrative modeling, and that setting this threshold was informed by the prior experience of our group. The RCSB PDB entry that matched the input protein most closely was then chosen and Clustal Omega v1.2.1 [33] was used to align the ATOM sequence of the template PDB file (i.e., the one describing homologous proteins of known structure) to the input protein sequence. Integrative models were subsequently generated using Modeller 9.16 [34]: ten models were built for each case and the one with the lowest Discrete Optimized Protein Energy score was chosen. The KiNG 2.21 [35] tool was used to visualize the generated 3D protein models.

## Results

### Genetic findings and clinical evaluation

Overall 181 probands with CC and/or anterior segment developmental anomalies (“CC group”) and 486 probands with RD (“RD group”) met the inclusion criteria for this study. In the CC group, 114 genes were analysed per case and a total of 11 small in-frame indels were detected in 12/181 study subjects. In the RD group, 176 genes were analysed per case and a total of 44 small in-frame indels were detected in 99/486 study subjects. Only one of these indels was detected in homozygous state, *CDHR1* c.690_692del. Notably, 17/55 (30.9 %) changes were novel to this study while 13/55 (23.6 %) variants were detected on multiple samples (range 2–21), and 35/55 (63.6 %) were found in a TR context. The mean and median number of affected amino acid residues was 2.2 and 1.5 respectively (range 1–7 amino acids as per definition of small indel used in this study). A detailed list of the identified variants can be found in Additional file 1: Table S2.

In terms of clinical evaluation, 5/11 changes from the CC group and 13/44 changes from the RD group were included in clinical reports; all remaining variants were included in technical reports. Genes in which clinically reported in-frame changes were identified include *BFSP2*, *CRYBA1*, *CRYBA4*, *CRYGC*, *PITX2, ABCA4*, *ADGRA3*, *CDHR1*, *CHM*, *CRB1*, *FLVCR1*, *INPP5E*, *NYX*, *PRPH2*, *RP2*, *RPE65* and *RS1*; a list of previously reported disease-associated small in-frame indels in these genes is shown in Additional file 1: Table S3. The predictions from all three computational tools used in this study (SIFT-indel, PROVEAN and DDG-in) were in agreement in 8/11 CC group variants and in 26/44 RD group variants. However, these predictions were not always in keeping with the conclusion in the clinical report. A notable example is the ABCA4 c.3840_3845del variant which was predicted neutral by all three tools but was reported to probably account for the clinical presentation in a 7-year-old study subject. This proband harbors another *ABCA4* change, c.1928G > T and has bilateral macular atrophy and yellow-white retinal lesions (flecks), a phenotype suggestive of *ABCA4*-retinopathy [36]. A second example is the *FSCN2* c.1071_1073del variant which was predicted to be damaging by all three *in silico* tools but was not considered likely to account for the clinical presentation in the affected proband. To date, the only reported link between *FSCN2* and retinal disease is a single bp deletion (rs376633374) that was identified in Japanese subjects with either retinitis pigmentosa [37] or macular dystrophy [38]. However, this variant did not segregate with retinal disease in Chinese families [39] and is unlikely to cause disease in a Mendelian fashion. Importantly, the proband, a 11-year-old subject with undetectable electroretinograms and an early-onset RD, also harbors a homozygous *GUCY2D* c.2285delG change. Biallelic GUCY2D changes are a common cause of early-onset RD and the c.2285delG change has been previously described in a 2-year-old affected individual [40]. Given the phenotype and the genetic findings it is much more likely that the condition is caused by recessive GUCY2D variants compared to dominant FSCN2 variants.

When integrative structural modeling was attempted, reliable models of the relevant protein sequences could be generated for 8/55 small in-frame indels (14.5 %; 5/11 in the CC group, 3/44 in the RD group) (Table 1).

**Table 1:** Small in-frame insertions/deletions for which reliable structural models could be generated

Gene	Sequence change	Protein change	Template used for integrative structural modeling	Structural modeling prediction: does this change disrupt protein structure/ function?	Clinical report: does this change account for the clinical presentation?	Is there agreement between *in silico* tools for this change?
*FSCN2*	c.1071_1073del	p.(Lys357del)	human FSCN1 (pdb 1DFC)	unlikely	unlikely	yes [D/D/D]
*RP2*	c.260_268del	p.(Thr87_Cys89del)	human RP2 (pdb 2BX6)	probably	possibly	yes [D/D/D]
*RPE65*	c.1443_1445del	p.(Glu481del)	cow RPE65 (pdb 3FSN)	unclear	probably	yes [D/D/D]
*BFSP2*	c.697_699del	p.(Glu233del)	human vimentin (pdb 3UF1)	probably	probably	yes [D/D/D]
*CRYBA1*	c.272_274del	p.(Gly91del)	human CRYBA4 (pdb 3LWK)	probably	probably	yes [D/D/D]
*CRYBA4*	c.136_156del	p.(Ser46_Gly52del)	human CRYBA4 (pdb 3LWK)	probably	probably	yes [D/D/D]
*CRYGC*	c.61_63del	p.(Thr21del)	human CRYGB (pdb 2JDF)	unclear	probably	yes [D/D/D]
*PITX2*	c.429_431del	p.(Arg144del)	human PITX2 (pdb 2LKX)	probably	probably	no [N/D/D]

Assuming the clinical report is the standard and after removing the case where the variant *possibly* accounted for the clinical presentation (RP2 p. (Thr87_Cys89del)), the test accuracy was found to be 0.86 for structural modeling, SIFT-indel and PROVEAN, and 0.71 for DDIG-in. SIFT-indel and PROVEAN had the highest sensitivity (1.00) while structural modeling had the highest specificity (0.75)

[D/D/D] suggests that an in-frame indel was predicted to be disease-associated by DDIG-in, damaging by SIFT-indel and deleterious by PROVEAN; [N/D/D] suggests that it was predicted to be neutral by DDG-in, damaging by SIFT-indel and deleterious by PROVEAN

For more details on transcripts, RCSB PDB entries, and *in silico* analysis please see text and Additional file 1: Table S2

### Integrative structural modeling in childhood cataract cases

**Fig. 1:**
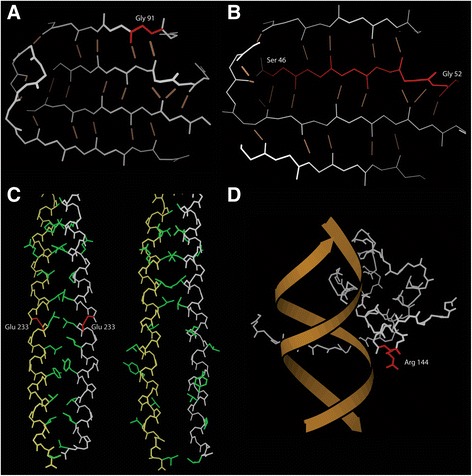
Integrative protein structure modeling for four variants identified in individuals with childhood cataracts. Affected amino acids are highlighted in red. **a**, **b** Models of the CRYBA1 c.272_274del, p. (Gly91del) (**a**) and CRYBA4 c.136_156del, p. (Ser46_Gly52del) (**b**) variants. The CRYBA1 and CRYBA4 proteins exhibit significant sequence similarity and the template with pdb code 3LWK (human β-crystallin A4) was used on both occasions. The main chain backbone atoms (*white*/*grey* lines) and the hydrogen bond network (*brown* lines) of the affected protein regions are shown. Both sequence alterations involve deleting residues located in β-sheets. **c** Homology model of the BFSP2 c.697_699del, p. (Glu233del) variant. BFSP2 forms parallel coiled-coil dimers that interact with one another in the form of a symmetrical anti-parallel dimer. The main chain backbone atoms (*white*/*yellow* lines) and the side chains that comprise the interaction interface (*green*) of the affected protein region are shown. The wild-type protein is presented on the left hand-side image. Notably, the affected amino acid is located in an α-helical region (highlighted in *red*). The right hand-side image shows a model of the mutant protein; the deletion shifts the position of the interacting side chains resulting in disruption of the dimer structure and exposure of the hydrophobic interface residues to the protein surface. **d** Model of the PITX2 c.429_431del, p. (Arg144del) variant. The main chain backbone atoms of the protein (*white*/*grey* lines) complexed with an interacting DNA double helix (*brown* chain) are shown. The mutated residue (highlighted in *red*) makes direct contact with the phosphate backbone of DNA, forming a salt bridge

In the majority of cases, simply highlighting the position of the indel on the protein structure gave a clear indication of its likely phenotypic effect. For both CRYBA1 c.272_274del and CRYBA4 c.136_156del variants the deleted residues are in β-sheets. The CRYBA1 change is a single residue deletion (Gly91) in an edge strand (Fig. 1a), whereas the CRYBA4 change is a larger deletion (Ser46_Gly52del) in a central strand (Fig. 1b). In general, β-sheet structures are highly constrained due to their hydrogen bond network [41] and so amino acid insertions and deletions are likely to be deleterious [42]. In conclusion, the CRYBA1 and CRYBA4 variants are likely to destabilise the corresponding proteins, leading to misfolding and aggregation. By contrast, the effect of the CRYGC c.61_63del variant is less clear as it removes an amino-acid (Thr21del) from a loop between two β-strands.

In the case of BFSP2 c.697_699del, the deleted residue (Glu233) is in the main α-helical region. In the wild-type, a long, continuous hydrophobic interface is formed between the protein chains (Fig. 1c, left hand-side image). Since there are 3.6 residues per turn in every α-helix, deletion of a single residue shifts the position of these hydrophobic residues from the internal interface to the surface of the protein (Fig. 1c, right hand-side image). The deletion is therefore likely to have two effects: firstly, the cognate interaction between the protein chains will be disrupted and secondly hydrophobic residues that are found on the surface of the protein in the mutant form will be able to form a wide array of non-cognate interactions, with the potential to form large aggregates.

For PITX2 c.429_431del, the deleted residue (Arg144) is in a surface loop, which, in general, is a structural context that is able to accommodate changes without substantially affecting protein folding. However, in the wild-type protein, Arg144 appears to make direct contact with the phosphate backbone of DNA forming a salt bridge (Fig. 1d). We therefore hypothesize that deletion of this residue would destabilise the protein-DNA interaction.

### Integrative structural modeling in retinal dystrophy cases

**Fig. 2:**
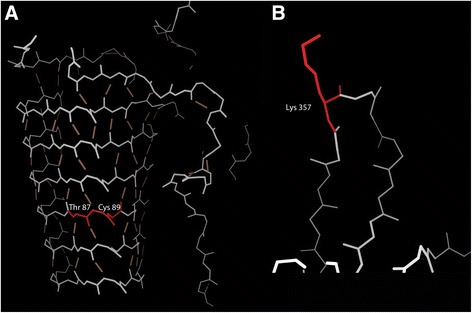
Integrative protein structure modeling for two variants identified in individuals with retinal dystrophy. Affected amino acids are highlighted in red. **a** Model of the RP2 c.260_268del, p. (Thr87_Cys89del) variant. The main chain backbone atoms (white/grey lines) and the hydrogen bond network (brown lines) of the affected protein region are shown. The variant is found in an extended set of β-sheets that form a complex set of hydrogen bonds. **b** Homology model of the FSCN2 c.1071_1073del, p. (Lys357del) variant. The main chain backbone atoms (white/grey lines) of a small part of the protein is shown. The deletion affects a residue in a surface loop, away from known functional or interaction sites

Indels in RD-associated genes offer useful contrasting examples. In RP2 c.260_268del the deleted residues (Thr87_Cys89) are found in a β-prism domain (Fig. 2a). Such an extended set of β-sheets is formed from cooperative sets of hydrogen bonds, and so any deletion is likely to be deleterious. By contrast, FSCN2 c.1071_1073del, leads to the deletion of Lys357 which is in a surface loop, away from known functional or interaction sites. This change is therefore unlikely to significantly disrupt protein structure or function. As discussed above, this deletion is predicted by SIFT-indel, PROVEAN and DDG-in to be deleterious, although it is unlikely to account for the clinical presentation. Therefore, in this case, structural analysis correlates more closely with clinical evaluation than sequence-based *in silico* tools.

The RPE65 c.1443_1445del change is more challenging to interpret. A negatively charged amino-acid (Glu481del) is removed resulting in loss of packing interactions that might contribute to the overall stability of the folded protein. However, the deletion appears to be away from catalytic/binding sites of the RPE65 enzyme, and commenting on variant pathogenicity on the basis of structural modeling would be highly speculative.

## Discussion

In this study we have investigated the role of small (≤21 bp) in-frame indels in two inherited eye disorders and have shown that integrative structural modeling can help interpret some of these changes. Known disease-associated genes were screened in 181 probands with CC and/or anterior segment developmental anomalies, and in 486 probands with RD; one small in-frame indel was clinically reported in 2.8 % (5/181) in 2.7 % (13/486) of cases respectively.

Although current high-throughput sequencing technologies provide unprecedented opportunities to detect genetic variation, it is still not possible to elucidate the molecular pathology in a significant proportion of cases with Mendelian disorders [43]. It has been previously shown that a genetic diagnosis cannot be identified in in 1 in 3 CC cases [44] and in 1 in 2 RD cases [16]. A combination of analytical/technical and biological factors are likely to contribute to this, including incomplete testing or knowledge of genes associated with these disorders [43]. One key factor is the inability of high-throughput sequencing to consistently and reliably detect indels [28]. There are two main reasons for this. First, most indels are associated with polymerase slippage and are located in difficult-to-sequence repetitive regions [30]. In the present study, we have not analysed 4 extremely repetitive exons (such as RPGR ORF15, see Additional file 1: Table S1) and we would therefore expect the true number of indel events to be higher. Second, numerous analytical/technical factors can affect indel detection accuracy including indel size, read coverage, read length and software tool options [28]. To minimize bias, we focused on small indels (≤21 bp), we analysed a high coverage subset (samples in which ≥99.5 % of target sequence had ≥50x coverage), and we employed the widely used Illumina chemistry (100 bp paired-end reads). Although there are bioinformatic pipelines that outperform the one utilized in this study [26–29, 45], at present, there is no gold standard method. It is noteworthy that the setting of this study is a clinical diagnostic laboratory and our findings reflect the current real-world diagnostic context.

To date, over 4000 disease-causing in-frame indels have been reported, corresponding to 2.2 % of all mutations (Human Gene Mutation Database, HGMD Professional release 2015.4). Recently, the 1000 Genomes Project Consortium reported that 1.4 % of detected exonic variants were indels [1] and it is expected that at least half of these changes will be in-frame [31]. Notably, functional and population annotations for these in-frame indels are becoming increasing available [1, 10]. In this study, three computational tools were used and their annotations were found to be in agreement for 61.8 % (34/55) of variants. However, the results were probably erroneous for at least two of these variants (*ABCA4* c.3840_3845del and the *FSCN2* c.1071_1073del). It can be speculated that the high degree of correlation between predictions (including the incorrect ones) was due to the fact that all three predictive models evaluated similar sets of variant properties (e.g. evolutionary conservation scores or regulatory-type annotations). We hypothesized that for the clinical utility to be maximised, not only the prediction but also the reasons for the prediction (e.g. disruption of a binding site or a β-sheet etc.) should be available to the clinician. Protein structure was therefore used as an endophenotype (defined by Karchin [11] as ‘measurable component unseen by the unaided eye along the pathway between disease and distal genotype’). Importantly only 1 in 7 in-frame indels were found within regions that could be reliably modeled. This mostly reflects the fact that integrative models often represent only fractions of the full-length of a protein [20]. Nevertheless, as new structures become available and new techniques are developed, the applicability and utility of the discussed methods is expected to grow.

A variety of properties can be evaluated to infer the impact of an amino acid sequence change on in vivo protein activity. Parameters assessed here and in previous studies include effect on protein folding/stability [46] and consequences on interaction interfaces [22]. Highly accurate protein structures are required for these types of analyses. To obtain such structures, we utilized a popular comparative modeling tool (Modeller 9.16 [34]). Notably, a range of similar tools has been described and objective testing/evaluation of these methods is regularly performed (see http://www.predictioncenter.org/). Although the pipeline and parameters used in this report have been carefully chosen, the current state of the art method remains to be established.

Structural analysis of mutant proteins in this study suggested that the abnormal phenotype can arise through diverse molecular mechanisms. These include alterations in the DNA interaction site of transcription factors (PITX2 c.429_431del), and disruption of secondary structural elements in crystallins (CRYBA1 c.272_274del, CRYBA4 c.136_156del), cytoskeletal constituents (BFSP2 c.697_699del) and GTPase-activating proteins (RP2 c.260_268del). This wide range of effects could only be rationalized with a combination of (i) careful clinical characterization, (ii) knowledge of the molecular and cellular function of the proteins in question, and (iii) modeling of the likely effects of indels in the context of protein structure and protein interactions. There is an acute need for computational tools that are able to estimate the relative pathogenicity of sequence variants of all types, including indels. Our findings suggest that if such tools are to be effective, they must be able to model the full complexity of molecular mechanisms by which pathogenicity arises.

## Conclusions

Systematic evaluation of the role of small in-frame indels in CC and RD revealed a clinically reported variant in every ~37 individuals tested for each group. Integrative structural modeling can be used to improve the diagnostic value of genetic testing in inherited eye disorders. The strategies presented have the potential to allow disease risk assessment at the atomic level, to facilitate study of multiple variant interactions (epistasis) and to guide knowledge-based interventions.

## Additional file

Additional file 1:
**Table S1.** Genes and transcripts included in multigene panel tests for retinal dystrophy and childhood cataracts. **Table S2.** Clinical and in silico evaluation of small (≤21 nucleotides) in-frame insertions/deletions identified by panel-based genetic diagnostic testing in 486 probands with retinal dystrophy and 181 probands with childhood cataract. **Table S3.** Previously reported disease-associated small in-frame insertions/deletions in genes found to have clinically reported variants in the present study. (PDF 241 kb)

## Abbreviations

3DEM: 3D electron microscopy
CC: Childhood cataracts
HR: Homopolymer runs
Indels: Insertions/deletions
MDT: Multidisciplinary team
NMR: Nuclear magnetic resonance
RD: Retinal dystrophies
SNVs: Single nucleotide variants
TR: Tandem repeats
UKAS - CPA: United Kingdom Accreditation Service - Clinical Pathology Accredited
VEP: Variant effect predictor
XRC: X-ray crystallography
