# Supplementary material for: The role of small in-frame insertions/deletions in inherited eye disorders and how structural modelling can help estimate their pathogenicity
Source: Orphanet J Rare Dis. 2016 Sep 14;11:125. doi: 10.1186/s13023-016-0505-0 (PMC5024463; doi:10.1186/s13023-016-0505-0)
Supplement: Additional file 1: — Table S1. Genes and transcripts included in multigene panel tests for retinal dystrophy and childhood cataracts. Table S2. Clinical and in silico evaluation of small (≤21 nucleotides) in-frame insertions/deletions identified by panel-based genetic diagnostic testing in 486 probands with retinal dystrophy and 181 probands with childhood cataract. Table S3. Previously reported disease-associated small in-frame insertions/deletions in genes found to have clinically reported variants in the present study. (PDF 241 kb) [file 13023_2016_505_MOESM1_ESM.pdf]

Table S1. Genes and transcripts included in multigene panel tests for retinal dystrophy and childhood cataracts

**RETINAL DYSTROPHY** (n=176)	
Gene (HGNC)	**NCBI RefSeq transcript (r73)**
ABCA4	NM_000350
ABHD12	NM_001042472
ACBD5	NM_145698
ADAM9	NM_003816
ADAMTS18	NM_199355
AHI1	NM_001134832; NM_017651
AIPL1	NM_014336
ARL2BP	NM_012106
ARL6	NM_032146
BBIP1	NM_001195306
BBS1	NM_024649
BBS10	NM_024685
BBS12	NM_001178007
BBS2	NM_031885
BBS4	NM_033028
BBS5	NM_152384
BBS7	NM_176824
BBS9	NM_198428
BEST1	NM_004183
C1QTNF5	NM_015645
C2orf71	NM_001029883
C8ORF37	NM_177965
C21orf2	NM_004928
CA4	NM_000717
CABP4	NM_145200
CACNA1F	NM_005183
CACNA2D4	NM_172364
CAPN5	NM_004055
CC2D2A	NM_001080522
CDH3	NM_001793
CDH23	NM_022124
CDHR1	NM_001171971; NM_033100
CEP164	NM_014956
CEP290	NM_025114
CERKL	NM_001030311
CHM	NM_000390
CIB2	NM_006383
CLN3	NM_000086
CLRN1	NM_052995; NM_001195794
CNGA1	NM_001142564
CNGA3	NM_001298
CNGB1	NM_001297
CNGB3	NM_019098
CNNM4	NM_020184
CRB1	NM_201253
CRX	NM_000554
CSPP1	NM_024790
CYP4V2	NM_207352
DFNB31	NM_015404
DHDDS	NM_024887
DTHD1	NM_001136536; NM_001170700
EFEMP1	NM_001039348
ELOVL4	NM_022726
EMC1	NM_015047
EYS	NM_001142800
FAM161A	NM_001201543
FLVCR1	NM_014053
FSCN2	NM_001077182
FZD4	NM_012193
GNAT1	NM_000172
GNAT2	NM_005272
GNPTG	NM_032520
GPR125	NM_145290
GPR179	NM_001004334
GPR98	NM_032119
GRM6	NM_000843
GUCA1A	NM_000409

**CHILDHOOD CATARACT** (n=114)	
Gene (HGNC)	**NCBI RefSeq transcript (r73)**
ADAMTS10	NM_030957.2
ADAMTSL4	NM_019032.4
AGK	NM_018238.3
AGPS	NM_003659.3
AKR1E2	NM_001040177.1
ALDH18A1	NM_002860.3
B3GALTL	NM_194318.3
BCOR	NM_017745.5; NM_001123385.1; NM_001123384.1
BFSP1	NM_001195.3
BFSP2	NM_003571.2
CBS	NM_001178008.1
CHMP4B	NM_176812.4
COL2A1	NM_001844.4
COL4A1	NM_001845.4
COL11A1	NM_001854.3
CRYAA	NM_000394.2
CRYAB	NM_001885.1
CRYBA1	NM_005208
CRYBA4	NM_001886.2
CRYBB1	NM_001887.3
CRYBB2	NM_000496.2
CRYBB3	NM_004076.3
CRYGC	NM_020989.3
CRYGD	NM_006891.3
CRYGS	NM_017541.2
CYP27A1	NM_000784.3
CYP51A1	NM_000786.3
DHCR7	NM_001360.2
EPHA2	NM_004431.3
ERCC2	NM_000400.3
ERCC3	NM_000122.1
ERCC6	NM_000124.2
ERCC8	NM_000082.3
EYA1	NM_172058.2; NM_172060.2
FAM126A	NM_032581.3
FBN1	NM_000138.4
FKRP	NM_001039885.2
FKTN	NM_006731.2
FOXC1	NM_001453.2
FOXD3	NM_012183.2
FOXE3	NM_012186.2
FTL	NM_000146.3
FYCO1	NM_024513.2
FZD4	NM_012193.3
GALK1	NM_000154.1
GALT	NM_000155.2
GCNT2	NM_145649.4; NM_145655.3; NM_001491.2
GJA1	NM_000165.3
GJA3	NM_021954.3
GJA8	NM_005267.4
GNPAT	NM_014236.3
HMX1	NM_018942.2
HSF4	NM_001040667.2
JAM3	NM_032801.3
LARGE	NM_004737.4
LMX1B	NM_001174146.1
*LRP5**	NM_002335.2
LTBP2	NM_000428.2
MAF	NM_005360.4
MAN2A	NM_002372.2.
MAN2B1	NM_000528
MFSD6L	NM_152599.3
MIP	NM_012064.3
MIR184	NR_029705.1
MYH9	NM_002473.4
NDP	NM_000266.3
NF2	NM_000268.3; NM_016418.5; NM_181832.2

**RETINAL DYSTROPHY** (n=176)	
Gene (HGNC)	**NCBI RefSeq transcript (r73)**
GUCA1B	NM_002098
GUCY2D	NM_000180
HARS	NM_002109
HMX1	NM_018942
IDH3B	NM_006899; NM_174855
IFT140	NM_014714
IMPDH1	NM_000883
IMPG1	NM_001563
IMPG2	NM_016247
INPP5E	NM_019892
INVS	NM_014425
IQCB1	NM_001023570
ITM2B	NM_021999
KCNJ13	NM_002242
KCNV2	NM_133497
KIAA1549	NM_001164665; NM_020910
KIF11	NM_004523
KLHL7	NM_001031710
LCA5	NM_181714
LRAT	NM_004744
*LRP5**	NM_002335
LZTFL1	NM_020347
MERTK	NM_006343
MFRP	NM_031433
MKKS	NM_018848
MKS1	NM_017777; NM_001165927
MVK	NM_000431
MYO7A	NM_000260
NDP	NM_000266
NEK2	NM_002497
NMNAT1	NM_0022787
NPHP1	NM_000272
NPHP3	NM_153240
NPHP4	NM_015102
NR2E3	NM_014249
NRL	NM_006177
NYX	NM_022567
OAT	NM_000274
OFD1	NM_003611
OTX2	NM_021728
PANK2	NM_153638
PCDH15	NM_001142763; NM_001142769; NM_001142770; NM_001142771
PCYT1A	NM_005017
PDE6A	NM_000440
PDE6B	NM_000283
PDE6C	NM_006204
PDE6G	NM_002602
PEX1	NM_000466
PEX2	NM_000318
PEX7	NM_000288
PHYH	NM_006214
PITPNM3	NM_031220
PLA2G5	NM_000929
PRCD	NM_001077620
PROM1	NM_006017
PRPF3	NM_004698
PRPF31	NM_015629
PRPF4	NM_004697
PRPF6	NM_012469
PRPF8	NM_006445
PRPH2	NM_000322
RAB28	NM_001017979
RAX2	NM_032753
RBP3	NM_002900
RBP4	NM_006744
RD3	NM_183059
RDH12	NM_152443
RDH5	NM_001199771
RGR	NM_002921

**CHILDHOOD CATARACT** (n=114)	
Gene (HGNC)	**NCBI RefSeq transcript (r73)**
NHS	NM_198270.2; NM_001136024.2
OCRL	NM_000276.3
OPA3	NM_001017989.2; NM_025136.3
PAX6	NM_001604.4
PEX1	NM_000466.2
PEX12	NM_000286.2
PEX13	NM_002618.3
PEX16	NM_057174.2; NM_004813.2
PEX2	NM_001172086.1
PEX26	NM_017929.5
PEX3	NM_003630.2
PEX6	NM_000287.3
PEX7	NM_000288.3
PEX5L	NM_016559.2
PEX10	NM_153818.1
*PEX11_*	NM_003846.2
PEX14	NM_004565.2
PEX19	NM_002857.3
PITX2	NM_000325.5; NM_153426.1
PITX3	NM_005029.3
POMT1	NM_007171.3
POMT2	NM_013382.5
PVRL3	NM_015480.1
PXDN	NM_012293.1
RAB18	NM_021252.3; NM_001256410
RAB3GAP1	NM_001172435.1
RAB3GAP2	NM_012414.3
RNLS	NM_001031709.2; NM_018363.3
SC5DL	NM_001024956.2
SEC23A	NM_006364.2
SIL1	NM_001037633.1
SIX5	NM_175875.4
SIX6	NM_007374.2
SLC16A12	NM_213606.3
SLC2A1	NM_006516.2
SLC33A1	NM_004733.3
SOLH	NM_005632.2
SOX2	NM_003106.3
SRD5A3	NM_024592.4
SREBF2	NM_004599.2
TDRD7	NM_014290.2
TFAP2A	NM_003220.2; NM_001042425.1; NM_001032280.2
TMEM70	NM_017866.5
TMEM114	NM_001146336.1
VIM	NM_003380.3
VSX2	NM_182894.2
WRN	NM_000553.4

RETINAL DYSTROPHY (n=176)	
Gene (HGNC)	**NCBI RefSeq transcript (r73)**
RGS9	NM_003835; NM_001165933
RHO	NM_000539
RIMS1	NM_014989; NM_001168407; NM_001168410
RLBP1	NM_000326
ROM1	NM_000327
RP1	NM_006269
*RP1L1**	NM_178857
RP2	NM_006915
RP9	NM_203288
RPE65	NM_000329
*RPGR**	NM_001034853
RPGRIP1	NM_020366
RPGRIP1L	NM_015272
RS1	NM_000330
SAG	NM_000541
SDCCAG8	NM_006642
SEMA4A	NM_022367
SLC24A1	NM_004727; NM_001254740
SNRNP200	NM_014014
SPATA7	NM_018418
TEAD1	NM_021961
TIMP3	NM_000362
TMEM237	NM_001044385
TOPORS	NM_005802
TRIM32	NM_012210
TRPM1	NM_002420
TSPAN12	NM_012338
TTC8	NM_144596
TUB	NM_177972
TULP1	NM_003322
UNC119	NM_005148; NM_054035
USH1C	NM_005709; NM_153676
USH1G	NM_173477
USH2A	NM_206933
VCAN	NM_004385
VPS13B	NM_017890
WDPCP	NM_015910
WDR19	NM_025132
ZNF423	NM_015069
ZNF513	NM_144631

* The following regions were not anaysed as they have little or no coverage due to technical limitations of the technology used: exon 1 of LRP5, exon 1 of MAF, exon 4 of RP1L1 and exon ORF15 of RPGR.

Table S2. Clinical and in silico evaluation of small (≤21 nucleotides) in-frame insertions/deletions identified by panel-based genetic diagnostic testing in 486 probands with retinal dystrophy and 181 probands with childhood cataract.

Presumed number of alleles	Gene (HGNC)	NCBI RefSeq transcript (r73)	Sequence change (Mutalyzer 2.0.15)	Protein change (Mutalyzer 2.0.15)	DDIG-in status [probability]	SIFT-indel effect [score]	PROVEAN prediction [score]	Previously reported (Ensembl r83)	Homopolymer run (HR) or tandem repeat (TR)	Status	Mode of inheritance associated with gene (OMIM)	Clinically reported ^
					**RETINAL DYSTROPHY** (176 genes evaluated; 486 individuals tested)							
2	ABCA4	NM_000350.2	c.3840_3845del	p.(Asp1281_Ser1282del)	N [80.10]	N [0.918]	N [-2.04]	rs62642572; see also Table S3	no HR/TR	het	AR	yes (probably accounts for clinical presentation)
1	ADGRA3	NM_145290.3	c.98_106del	p.(Gly33_Gly35del)	N [86.40]	N [0.696]	N [0.94]	no	TR	het	AR	yes (unknown clinical significance)
8	ADGRA3	NM_145290.3	c.104_106del	p.(Gly35del)	N [94.80]	N [0.696]	N [1.43]	rs769005255	TR	unknown	AR	no (unknown clinical significance)
5	C2orf71	NM_001029883.2	c.3264_3266del	p.(Pro1089del)	N [89.50]	N [0.696]	D [-9.33]	rs138020654	no HR/TR	unknown	AR	no (unknown clinical significance)
1	C1QTNF5	NM_015645.3	c.163_171dup	p.(Arg55_Gly57dup)	N [69.30]	D [0.943]	N [-1.75]	no	TR	het	AD	no (unknown clinical significance)
11	CACNA1F	NM_005183.3	c.2442_2444dup	p.(Glu825dup)	N [91.50]	N [0.696]	N [-0.89]	rs112450928	TR	unknown	XL	no (unknown clinical significance)
1	CACNA1F	NM_005183.3	c.2457_2474dup	p.(Glu820_Glu825dup)	N [80.60]	N [0.696]	N [0.59]	no	TR	het	XL	no (unknown clinical significance)
21	CC2D2A	NM_001080522.2	c.685_687del	p.(Glu229del)	N [89.40]	N [0.696	N [-0.76]	rs386833764, rs112367037	TR	unknown	AR	no (unknown clinical significance)
1	CDHR1	NM_033100.3	c.690_692del	p.(Thr231del)	D [83.20]	D [0.894]	D [-5.96]	no	TR	hom	AR	yes (possibly accounts for clinical presentation)
1	CEP164	NM_014956.4	c.799_801del	p.(Lys267del)	N [90.20]	D [0.667]	N [-0.40]	rs764638875	TR	unknown	AR	no (unknown clinical significance)
1	CEP164	NM_014956.4	c.3934_3939dup	p.(Pro1312_Thr1313dup)	N [92.00]	D [0.667]	N [-1.25]	rs749695145	TR	unknown	AR	no (unknown clinical significance)
1	CHM	NM_000390.2	c.1922_1933dup	p.(Asn644_Leu645insGlnSerThrAsn)	N [87.30]	D [0.667]	N [-0.82]	rs767043768	no HR/TR	hemi	XL	yes (possibly accounts for clinical presentation)
4	CNGB1	NM_001297.4	c.1101_1103dup	p.(Glu371dup)	N [89.30]	N [0.696]	N [-0.74]	rs374653770, rs753335204	TR	unknown	AR	no (unknown clinical significance)
1	CNGB1	NM_001297.4	c.1659_1670del	p.(Ala554_Ala557del)	N [77.00]	N [0.918]	D [-15.06]	rs769138379	no HR/TR	unknown	AR	no (unknown clinical significance)
1	CNGB3	NM_019098.4	c.2179_2199del	p.(Gln727_Lys733del)	N [87.30]	N [0.696]	N [1.46]	rs746549330	no HR/TR	unknown	AR	no (unknown clinical significance)
1	CNNM4	NM_020184.3	c.2203_2211del	p.(Gly735_Thr737del)	N [86.60]	N [0.918]	D [-2.73]	rs550956407	no HR/TR	unknown	AR	no (unknown clinical significance)
3	CRB1	NM_201253.2	c.498_506del	p.(Ile167_Gly169del)	D [84.80]	D [0.894]	D [-14.26]	rs748136623	no HR/TR	het	AR,AD	yes (probably accounts for clinical presentation)
1	DFNB31	NM_015404.3	c.376_378del	p.(Ala126del)	N [89.00]	D [0.765]	N [-1.08]	rs777938907	no HR/TR	unknown	AR	no (unknown clinical significance)
1	EYS	NM_001142800.1	c.8427_8432del	p.(Thr2810_Lys2811del)	D [58.40]	N [0.636]	D [-4.10]	no	no HR/TR	het	AR	no (unknown clinical significance)
1	FLVCR1	NM_014053.3	c.774_779dup	p.(Leu259_Val260dup)	D [85.60]	D [0.943]	D [-9.97]	rs749580355	no HR/TR	het	AR	yes (possibly accounts for clinical presentation)
1	FSCN2	NM_001077182.2	c.1071_1073del	p.(Lys357del)	D [87.20]	D [0.894]	D [-12.50]	rs760309853	TR	het	AD	no (unknown clinical significance)
2	GUCY2D	NM_000180.3	c.129_134del	p.(Leu44_Leu45del)	N [54.30]	N [0.918]	N [-1.78]	rs552184470	no HR/TR	unknown	AR, AD	no (unknown clinical significance)
1	INPP5E	NM_019892.4	c.560_561insGCTGCCCAT	p.(Ser187delinsArgLeuProIle)	N [89.90]	N [0.918]	D [-3.44]	rs769234632	TR	het	AR	yes (does not account for clinical presentation)
1	IFT140	NM_014714.3	c.3955_3960del	p.(Ala1319_Lys1320del)	D [77.90]	D [0.667]	D [-4.36]	rs746697405	TR	unknown	AR	no (unknown clinical significance)
1	NPHP4	NM_015102.4	c.1306_1314dup	p.(Lys436_Val438dup)	N [81.80]	n/a	D [-5.49]	rs747743472	TR	unknown	AR	no (unknown clinical significance)
1	MYO7A	NM_000260.3	c.6614_6634dup	p.(Met2205_Ser2211dup)	D [95.40]	D [0.667]	D [-6.14]	rs111033388	no HR/TR	unknown	AR	no (unknown clinical significance)
1	NRL	NM_006177.3	c.366_368del	p.(Ala123del)	N [92.60]	N [0.918]	D [-2.65]	no	no HR/TR	het	AD,AR	no (unknown clinical significance)
1	NYX	NM_022567.2	c.106_111del	p.(Ala36_Cys37del)	D [92.70]	D [0.894]	D [-7.77]	no	TR	hemi	XL	yes (unknown clinical significance)
1	OFD1	NM_003611.2	c.2467_2469del	p.(Glu823del)	N [93.20]	N [0.696]	D [-4.81]	rs771984968	TR	unknown	AR	no (unknown clinical significance)
2	PCDH15	NM_001142763.1	c.5275_5277del	p.(Pro1759del)	N [93.40]	N [0.696]	N [0.47]	rs397517462	TR	uknown	AR	no (unknown clinical significance)
1	PCDH15	NM_001142763.1	c.5308_5313del	p.(Ala1770_Pro1771del)	N [88.50]	D [0.667]	N [-0.65]	rs397517465	TR	uknown	AR	no (unknown clinical significance)
1	PCDH15	NM_001142763.1	c.5390_5395del	p.(Leu1797_Pro1798del)	N [90.30]	N [0.696]	N [-0.72]	rs779691085	TR	uknown	AR	no (unknown clinical significance)
5	PCDH15	NM_001142763.1	c.5622_5624del	p.(Thr1876del)	N [93.20]	N [0.696]	N [-0.09]	rs113363047	TR	unknown	AR	no (unknown clinical significance)
1	PRPH2	NM_000322.4	c.603_620del	p.(Arg203_Gly208del)	D [97.40]	D [0.894]	D [-28.17]	no	no HR/TR	het	AD,AR	yes (probably accounts for clinical presentation)
1	RAX2	NM_032753.3	c.417_422dup	p.(Pro140_Gly141dup)	N [86.30]	D [0.667]	D [-3.59]	rs549932754	TR	unknown	AD	no (unknown clinical significance)
1	RP2	NM_006915.2	c.260_268del	p.(Thr87_Cys89del)	D [97.50]	D [0.894]	D [-22.94]	no	no HR/TR	hemi	XL	yes (possibly accounts for clinical presentation)
1	RPE65	NM_000329.2	c.1443_1445del	p.(Glu481del)	D [87.20]	D [0.894]	D [-11.24]	no	TR	het	XL	yes (probably accounts for clinical presentation)
1	RS1	NM_000330.3	c.496_498del	p.(Tyr166del)	D [85.00]	D [0.894]	D [-9.84]	CD136241; see also Table S3	TR	hemi	XL	yes (probably accounts for clinical presentation)
1	RS1	NM_000330.3	c.592_600dup	p.(Phe198_Arg200dup)	D [94.30]	D [0.894]	D [-11.00]	no	no HR/TR	hemi	XL	yes (probably accounts for clinical presentation)
1	SLC24A1	NM_004727.2	c.2071_2073del	p.(Lys691del)	N [93.00]	N [0.918]	N [-0.34]	rs748047300	TR	unknown	AR	no (unknown clinical significance)
2	SLC24A1	NM_004727.2	c.2658_2660del	p.(Glu890del)	N [91.50]	D [0.667]	N [-0.46]	rs765607758	TR	unknown	AR	no (unknown clinical significance)
2	TULP1	NM_003322.4	c.371_394del	p.(Asp124_Glu131del)	N [85.30]	N [0.918]	D [-5.59]	rs281865169, rs558243084	TR	unknown	AR	no (unknown clinical significance)
1	VCAN	NM_004385.4	c.4183_4185del	p.(Glu1395del)	N [90.80]	N [0.918]	D [-2.01]	rs758450587	TR	het	AD	no (unknown clinical significance)
2	VPS13B	NM_017890.4	c.8833_8834insGAA	p.(Pro2945delinsArgThr)	N [63.60]	D [0.765]	D [-6.17]	rs758685660	no HR/TR	unknown	AR	no (unknown clinical significance)
					**CHILDHOOD CATARACT** (114 genes evaluated; 181 individuals tested)							
1	BFSP2	NM_003571.3	c.697_699del	p.(Glu233del)	D [88.50]	D [0.943]	D [-14.08]	rs121908938; see also Table S3	TR	het	AD	yes (probably accounts for clinical presentation)
1	CAPN15	NM_005632.2	c.376_381del	p.(Lys126_Asp127del)	N [87.60]	N [0.918]	N [-1.56]	rs771271054	no HR/TR	unknown	AD	no (unknown clinical significance)
1	CRYBA1	NM_005208.4	c.272_274del	p.(Gly91del)	D [87.60]	D [0.894]	D [-15.88]	CD040165; see also Table S3	TR	het	AD	yes (probably accounts for clinical presentation)
1	CRYBA4	NM_001886.2	c.136_156del	p.(Ser46_Gly52del)	D [98.80]	D [0.894]	D [-29.50]	no	no HR/TR	het	AD	yes (probably accounts for clinical presentation)
1	CRYGC	NM_020989.3	c.61_63del	p.(Thr21del)	D [88.40]	D [0.846]	D [-8.17]	no	no HR/TR	het	AD	yes (probably accounts for clinical presentation)
1	FOXC1	NM_001453.2	c.1139_1141del	p.(Gly380del)	N [91.10]	N [0.696]	N [-0.50]	rs751183793	TR	het	AD	no (unknown clinical significance)
2	FOXC1	NM_001453.2	c.1359_1361dup	p.(Gly456dup)	N [91.10]	D [0.667]	N [-0.88]	rs572346201	TR	het	AD	no (unknown clinical significance)
1	FOXC1	NM_001453.2	c.1476_1481del	p.(Ala494_Ala495del)	N [83.40]	N [0.696]	N [-1.86]	rs771540524	TR	het	AD	no (unknown clinical significance)
1	MAF	NM_001031804.2	c.696_701dup	p.(Gly237_Gly238dup)	N [89.00]	N [0.696]	N [-0.86]	no	TR	het	AD	no (unknown clinical significance)
1	NHS	NM_198270.2	c.216_218del	p.(Pro73del)	N [92.70]	D [0.667]	N [-0.49]	rs10590816	TR	hemi	XL	no (unknown clinical significance)
1	PITX2	NM_000325.5	c.429_431del	p.(Arg144del)	N [62.40]	D [0.943]	D [-13.92]	no	TR	het	AD	yes (probably accounts for clinical presentation)

N corresponds to neutral (colour-coded in dark green), D corresponds to disease, damaging or deleterious (colour-coded in dark red).

Het, hom and hemi corresponds to heterozygous, homozygous and hemizygous; in variants with 'unknown' status, the zygosity was not confirmed by Sanger sequencing.

HR corresponds to homopolymer run (i.e. the variant is within a run of six or more identical bases) and TR corresponds to tandem repeat (i.e. the variant is within a segment of at least two repeated sequences).

AR, AD and XL correspond to autosomal recessive, autosomal dominant or X-linked recessive mode of inheritance.

^ Clinically reported variants are coded in red ('probably accounts for clinical presentation'), light orange ('possibly accounts for clinical presentation') or grey ('unknown clinical significance' or 'does not account for clinical presentation'). Variants that were not clinically reported are coded in light green.

Table S3. Previously reported disease-associated small in-frame insertions/deletions in genes found to have clinically reported variants in the present study

Gene	Sequence change	Protein change	First report
BFSP2	c.697_699delGAA	p.(Glu233del)	1 †
CRYBA1	c.272_274del	p.(Gly91del)	2 †
ABCA4	c.38_46del	p.(Lys13_Trp15del)	3
ABCA4	c.1506_1514del	p.(Phe503_Ile505del)	4
ABCA4	c.2183_2194del	p.(Ser728_Phe731del)	5
ABCA4	c.2291_2305del	p.(Cys764_Ile768del)	6
ABCA4	c.2625_2626insTTT	p.(Leu875_Gln876insPhe)	7
ABCA4	c.3449_3451del	p.(Cys1150del)	8
ABCA4	c.3840_3845del	p.(Asp1281_Ser1282del)	9 †
ABCA4	c.4249_4251delTTC	p.(Phe1417del)	10
ABCA4	c.4735_4739delinsCC	p.(Phe1579_Leu1580delinsPro)	11
ABCA4	c.5216_5218dup	p.(Ala1739dup)	12
ABCA4	c.5281_5289del	p.(Pro1761_Leu1763del)	13
ABCA4	c.5668_5670del	p.(Phe1890del)	14
CDHR1	c.1311_1316del	p.(Leu437_Thr438del)	15
CHM	c.872_880del	p.(Glu291_Met294delinsVal)	16
CRB1	c.498_506del	p.(Ile167_Gly169del)	17
CRB1	c.2245_2247del	p.(Ser749del)	18
CRB1	c.2365_2367del	p.(Asn789del)	19
CRB1	c.2886_2888del	p.(Leu962del)	18
NYX	c.302_304del	p.(Ile101del)	20
NYX	c.339_353del	p.(Glu114_Ala118del)	21
NYX	c.340_354del	p.(Glu114_Ala118del)	20
NYX	c.619_627dup	p.(Arg207_Arg209dup)	20
NYX	c.620_628dup	p.(Arg209_Ser210insCysLeuArg)	22
NYX	c.732_743del	p.(Glu244_Ala247del)	20
NYX	c.1370_1387del	p.(Gln457_Ala463delinsPro)	23
PRPH2	c.198_202delinsAAGA CACA	p.(Met67_Gly68delinsArgHisArg)	24
PRPH2	c.199_201del	p.(Met67del)	25
PRPH2	c.356_358del	p.(Cys119del)	26
PRPH2	c.461_463del	p.(Lys154del)	27
PRPH2	c.505_507del	p.(Asn169del)	28
PRPH2	c.577_579del	p.(Lys193del)	25
PRPH2	c.616_627del	p.(Val206_Val209del)	29
PRPH2	c.618_626del	p.(Asp207_Val209del)	30
PRPH2	c.656_658del	p.(Pro219del)	31
PRPH2	c.711_722del	p.(Asp237_Thr240del)	32
PRPH2	c.811_813del	p.(Leu271del)	33
PRPH2	c.914_922del	p.(Gly305_Leu308delinsVal)	34

Gene	Sequence change	Protein change	First report
RP2	c.14_16del	p.(Phe5del)	35
RP2	c.16_18del	p.(Ser6del)	36
RP2	c.409_411del	p.(Ile137del)	37
RPE65	c.106_114del	p.(Leu36_Leu38del)	38
RPE65	c.991_993dup	p.(Trp331dup)	39
RPE65	c.1046_1047insTGG	p.(Asn349_Trp350insGly)	40
RS1	c.253_255del	p.(Asn85del)	41
RS1	c.306_308dup	p.(Leu103dup)	42
RS1	c.327_329del	p.(Cys110del)	43
RS1	c.496_498del	p.(Tyr166del)	44 †

The following genes were investigated for previously reported disease-associated small in-frame insertions/deletions (indels): *BFSP2, CRYBA1, CRYBA4, CRYGC, PITX2, ABCA4, ADGRA3, CDHR1, CHM, CRB1, FLVCR1, INPP5E, NYX, PRPH2, RP2, RPE65* and *RS1*. These were selected as they were the genes in which clinically reported small in-frame indels were identified in the studied cohort (see text for more information). The Human Gene Mutation Database (HGMD Professional release 2016.2) and Ensembl Release 83 datasets were inspected (accessed 28 Jul 2016). No disease-associated small in-frame indels were detected in *CRYBA4, CRYGC, PITX2, ADGRA3, FLCVR1* and *INPP5E*. For information on transcripts used please refer to Table S2.
† suggests that the variant was also identified in the present cohort .

REFERENCES

1. Jakobs PM, Hess JF, FitzGerald PG, Kramer P, Weleber RG, Litt M: Autosomal-dominant congenital cataract associated with a deletion mutation in the human beaded filament protein gene BFSP2. Am J Hum Genet 2000; 66: 1432-1436.
2. Qi Y, Jia H, Huang S, et al: A deletion mutation in the betaA1/A3 crystallin gene (CRYBA1/A3) is associated with autosomal dominant congenital nuclear cataract in a Chinese family. Hum Genet 2004; 114: 192-197.
3. Briggs CE, Rucinski D, Rosenfeld PJ, Hirose T, Berson EL, Dryja TP: Mutations in ABCR (ABCA4) in patients with Stargardt macular degeneration or cone-rod degeneration. Invest Ophthalmol Vis Sci 2001; 42: 2229-2236.
4. Jaakson K, Zernant J, Külm M, et al: Genotyping microarray (gene chip) for the ABCR (ABCA4) gene. Hum Mutat 2003; 22: 395-403.
5. Simonelli F, Testa F, Zernant J, et al: Genotype-phenotype correlation in Italian families with Stargardt disease. Ophthalmic Res 2005; 37: 159-167.
6. Chen Y, Ratnam K, Sundquist SM, et al: Cone photoreceptor abnormalities correlate with vision loss in patients with Stargardt disease. Invest Ophthalmol Vis Sci. 2011; 52: 3281-3292.
7. Testa F, Rossi S, Sodi A, et al: Correlation between photoreceptor layer integrity and visual function in patients with Stargardt disease: implications for gene therapy. Invest Ophthalmol Vis Sci 2012; 53: 4409-4415.

8. Beit-Ya'acov A, Mizrahi-Meissonnier L, Obolensky A, et al: Homozygosity for a novel ABCA4 founder splicing mutation is associated with progressive and severe Stargardt-like disease. Invest Ophthalmol Vis Sci 2007 ; 48:4308-4314.
9. Webster AR, Héon E, Lotery AJ, et al: An analysis of allelic variation in the ABCA4 gene. Invest Ophthalmol Vis Sci 2001; 42: 1179-1189.
10. Chacón-Camacho OF, Granillo-Alvarez M, Ayala-Ramírez R, Zenteno JC: ABCA4 mutational spectrum in Mexican patients with Stargardt disease: Identification of 12 novel mutations and evidence of a founder effect for the common p.A1773V mutation. Exp Eye Res 2013; 109: 77-82.
11. Stenirri S, Alaimo G, Manitto MP, Brancato R, Ferrari M, Cremonesi L: Are microarrays useful in the screening of ABCA4 mutations in Italian patients affected by macular degenerations? Clin Chem Lab Med 2008; 46: 1250-1255.
12. Fujinami K, Zernant J, Chana RK, et al: Clinical and molecular characteristics of childhood-onset Stargardt disease. Ophthalmology 2015; 122: 326-334.
13. Lewis RA, Shroyer NF, Singh N, et al: Genotype/Phenotype analysis of a photoreceptor-specific ATP-binding cassette transporter gene, ABCR, in Stargardt disease. Am J Hum Genet 1999; 64: 422-434.
14. Maugeri A, van Driel MA, van de Pol DJ, et al: The 2588G>C mutation in the ABCR gene is a mild frequent founder mutation in the Western European population and allows the classification of ABCR mutations in patients with Stargardt disease. Am J Hum Genet 1999; 64: 1024-1035.
15. Glöckle N, Kohl S, Mohr J, et al: Panel-based next generation sequencing as a reliable and efficient technique to detect mutations in unselected patients with retinal dystrophies. Eur J Hum Genet 2014; 22: 99-104.
16. Ramsden SC, O'Grady A, Fletcher T, et al: A clinical molecular genetic service for United Kingdom families with choroideraemia. Eur J Med Genet 2013; 56: 432-438.
17. Vallespin E, Cantalapiedra D, Garcia-Hoyos M, et al: Gene symbol: CRB1. Disease: Leber congenital amaurosis. Accession #Hd0510. Hum Genet 2006; 118: 774.
18. Bernal S, Calaf M, Garcia-Hoyos M, et al: Study of the involvement of the RGR, CRPB1, and CRB1 genes in the pathogenesis of autosomal recessive retinitis pigmentosa. J Med Genet 2003; 40: e89.
19. Bujakowska K, Audo I, Mohand-Saïd S, et al: CRB1 mutations in inherited retinal dystrophies. Hum Mutat 2012; 33: 306-315.
20. Pusch CM, Zeitz C, Brandau O, et al: The complete form of X-linked congenital stationary night blindness is caused by mutations in a gene encoding a leucine-rich repeat protein. Nat Genet 2000; 26: 324-327.
21. Zito I, Allen LE, Patel RJ, et al: Mutations in the CACNA1F and NYX genes in British CSNBX families. Hum Mutat 2003; 21: 169.
22. Bech-Hansen NT, Naylor MJ, Maybaum TA, et al: Mutations in NYX, encoding the leucine-rich proteoglycan nyctalopin, cause X-linked complete congenital stationary night blindness. Nat Genet 2000; 26: 319-323.
23. Zeitz C, Labs S, Lorenz B, et al: Genotyping microarray for CSNB-associated genes. Invest Ophthalmol Vis Sci 2009; 50: 5919-5926.

24. Jacobson SG, Cideciyan AV, Kemp CM, Sheffield VC, Stone EM: Photoreceptor function in heterozygotes with insertion or deletion mutations in the RDS gene. Invest Ophthalmol Vis Sci 1996; 37: 1662-1674.
25. Jacobson SG, Kemp CM, Cideciyan AV, et al: Spectrum of functional phenotypes in RDS gene mutations. Invest Ophthalmol Vis Sci 1994; 35: 1479A.
26. Farrar GJ, Kenna P, Jordan SA, et al: A three-base-pair deletion in the peripherin-RDS gene in one form of retinitis pigmentosa. Nature 1991; 354: 478-480.
27. Weleber RG, Carr RE, Murphey WH, Sheffield VC, Stone EM: Phenotypic variation including retinitis pigmentosa, pattern dystrophy, and fundus flavimaculatus in a single family with a deletion of codon 153 or 154 of the peripherin/RDS gene. Arch Ophthalmol 1993; 111: 1531-1542.
28. van Lith-Verhoeven JJ, van den Helm B, Deutman AF, et al: A peculiar autosomal dominant macular dystrophy caused by an asparagine deletion at codon 169 in the peripherin/RDS gene. Arch Ophthalmol 2003; 121: 1452-1457.
29. Gannon AM, Rodriguez JA, Humphries P, Birch DG, Heckenlively JR, Daiger SP: Mutations in peripherin/RDS in patients with retinitis pigmentosa: a 12 base-pair deletion in exon 2. Am J Hum Genet 1993 (Suppl.).
30. Kalyanasundaram TS, Black GC, O'Sullivan J, Bishop PN: A novel peripherin/RDS mutation resulting in a retinal dystrophy with phenotypic variation. Eye (Lond) 2009; 23: 237-239.
31. Kajiwara K, Hahn LB, Mukai S, Travis GH, Berson EL, Dryja TP: Mutations in the human retinal degeneration slow gene in autosomal dominant retinitis pigmentosa. Nature 1991; 354: 480-483.
32. Gamundi MJ, Hernan I, Muntanyola M, et al: High prevalence of mutations in peripherin/RDS in autosomal dominant macular dystrophies in a Spanish population. Mol Vis 2007; 13: 1031-1037.
33. Jin ZB, Mandai M, Yokota T, et al: Identifying pathogenic genetic background of simplex or multiplex retinitis pigmentosa patients: a large scale mutation screening study. J Med Genet 2008; 45: 465-472.
34. Heinzmann C, Kojis TL, Flodman P, et al: Novel nine base-pair deletion in the third exon of RDS/peripherin causative of autosomal dominant retintis pigmentosa in a large Mexican family (UCLA-RP12). Am J Hum Genet. 1995; 57: A214.
35. Neidhardt J, Glaus E, Lorenz B, et al: Identification of novel mutations in X-linked retinitis pigmentosa families and implications for diagnostic testing. Mol Vis 2008; 14: 1081-1093.
36. Schwahn U, Lenzner S, Dong J, et al: Positional cloning of the gene for X-linked retinitis pigmentosa 2 Nat Genet 1998; 19: 327-332.
37. Sharon D, Bruns GA, McGee TL, et al: X-linked retinitis pigmentosa: mutation spectrum of the RPGR and RP2 genes and correlation with visual function. Invest Ophthalmol Vis Sci 2000; 41: 2712-2721.
38. Sitorus RS, Lorenz B, Preising MN: Analysis of three genes in Leber congenital amaurosis in Indonesian patients. Vision Res 2003; 43: 3087-3093.

39. Coppieters F, Casteels I, Meire F, et al: Genetic screening of LCA in Belgium: predominance of CEP290 and identification of potential modifier alleles in AHI1 of CEP290-related phenotypes. Hum Mutat 2010; 31: E1709-1766.
40. Hamel CP, Griffoin JM, Bazalgette C, et al: [Molecular genetics of pigmentary retinopathies: identification of mutations in CHM, RDS, RHO, RPE65, USH2A and XLRS1 genes]. J Fr Ophtalmol 2000; 23: 985-995.
41. Hiriyanna KT, Bingham EL, Yashar BM, et al: Novel mutations in XLRS1 causing retinoschisis, including first evidence of putative leader sequence change. Hum Mutat 1999; 14: 423-427.
42. Bowles K, Cukras C, Turriff A, et al: X-linked retinoschisis: RS1 mutation severity and age affect the ERG phenotype in a cohort of 68 affected male subjects. Invest Ophthalmol Vis Sci 2011; 52: 9250-9256.
43. Hewitt AW, FitzGerald LM, Scotter LW, Mulhall LE, McKay JD, Mackey DA: Genotypic and phenotypic spectrum of X-linked retinoschisis in Australia. Clin Experiment Ophthalmol 2005; 33: 233-239.
44. Vincent A, Robson AG, Neveu MM, et al. A phenotype-genotype correlation study of X-linked retinoschisis. Ophthalmology 2013; 120: 1454-1464.
